# The Association Between Changes in White Matter Microstructure and Cognitive Function in Older Adults with Mild Cognitive Impairment

**DOI:** 10.3390/brainsci16060655

**Published:** 2026-06-22

**Authors:** Yuehong Qiu, Can Jiao

**Affiliations:** 1 College of Psychology, Liaoning Normal University, Dalian 116082, China; 18565643812@163.com; 2 School of Government, Shenzhen University, Shenzhen 518060, China

**Keywords:** mild cognitive impairment, Cognitive Function Measurement Scale for the Elderly, fractional anisotropy, Mean Diffusivity, white matter microstructure

## Abstract

**Background**: Mild Cognitive Impairment (MCI) is a clinical state between normal aging and dementia. It may involve impairment in one or several cognitive domains. MCI offers a key window for maintaining cognitive function and studying how deficits develop in the elderly, making it of great research value. Measurement tools for screening MCI are not yet standardized in China. The accuracy of diagnostic criteria and threshold values needs improvement. Previous studies on the neural mechanisms of MCI have examined various aspects, but the changes in the white matter microstructure in older adults with MCI remain unclear. Most past studies used Fractional Anisotropy (FA) analysis to examine changes in white matter fiber orientation, often ignoring fiber density. As a result, findings are often contradictory or difficult to interpret. Therefore, it is necessary to assess cognitive function in MCI populations using more comprehensive and standardized measurement tools. It is also important to explore the association between changes in white matter microstructure and cognitive function in MCI by analyzing FA and Mean Diffusivity (MD). **Methods**: First, we assessed cognitive function using the Cognitive Function Measurement Scale for the Elderly, developed by Beijing Normal University, with diagnoses based on the NIA-AA (National Institute on Aging—Alzheimer’s Association) criteria. Second, we employed Diffusion Tensor Imaging (DTI) combined with Tract-Based Spatial Statistics (TBSS) to investigate alterations in the white matter fiber tract integrity in individuals with MCI. Based on the metrics used, this study was divided into two analytical approaches: Analysis Mode 1 utilized FA to explore changes in white matter fiber orientation in the MCI group. Analysis Mode 2 utilized MD to examine changes in white matter fiber density in the MCI group. Third, we further explored the association between alterations in the white matter fiber tract integrity and cognitive function in individuals with MCI. Specifically, FA and MD values from brain regions showing significant differences between the MCI and normal control groups were extracted and correlated with cognitive test scores. **Results**: According to the results of the community measurement survey, the prevalence of MCI among the elderly in Shenzhen is approximately 21.54%. Individuals with MCI exhibited functional decline in memory, attention, language, executive function, and spatial processing. DTI results indicated that (1) FA values across the brain’s white matter fiber tracts showed a decreasing trend in the elderly with MCI, with no areas exhibiting significantly higher FA values. Specifically, FA values were significantly lower in the corpus callosum, internal capsule, corona radiata, thalamic radiation, external capsule, superior fronto-occipital fasciculus, and cingulum (cingulate gyrus). (2) White matter fiber tracts with significantly reduced FA values also demonstrated significantly increased MD values. Additionally, MD values in the cingulum (hippocampus), inferior cerebellar peduncle, and corticospinal tract were significantly reduced in the MCI group. (3) Correlation analysis revealed that the significant differences in FA and MD values within the white matter fiber tracts of older adults with MCI were correlated with scores on several cognitive tests. **Conclusions**: In the present study, older adults with MCI tended to exhibit functional decline across multiple cognitive domains and relatively extensive microstructural white matter damage. Observations suggested that white matter fiber density may be informative regarding these microstructural alterations, indicating that diffusion biomarkers in key regions such as the cingulum (hippocampus) warrant further investigation.

## 1. Introduction

Mild Cognitive Impairment (MCI) is a clinical state intermediate between normal aging and dementia, primarily characterized by memory loss that exceeds what is typical for age and education level, often accompanied by mild impairment in other cognitive domains [1,2]. In contrast to the generally irreversible nature of Alzheimer’s disease (AD), MCI represents a critical “window period” in the progression of cognitive impairment. Therefore, timely detection and effective intervention for MCI are key to treating cognitive deficits and slowing or preventing progression to AD [3], highlighting the significant clinical and research value of MCI.

Cognitive functions such as memory, language, attention, and executive function generally deteriorate with age in older adults with MCI. However, current instruments for measuring cognitive function in MCI vary considerably in their systematicity and domain coverage. Moreover, variations in measurement scales, evaluation criteria, and threshold definitions have led to inconsistent results across related studies.

The National Institute on Aging (NIA) and the Alzheimer’s Association (AA) updated and expanded the diagnostic criteria for pre-Alzheimer’s disease based on Petersen’s work. These criteria are now widely recognized as the standard for diagnosing MCI [4]. Building on Albert et al.’s (2011) [4] research and using the NIA-AA diagnostic criteria as a blueprint, Beijing Normal University (BNU) developed the Cognitive Function Measurement Scale for the Elderly (CFMSE) and adapted it to the Chinese cultural context. The CFMSE categorizes cognitive function into five domains: memory, language, attention, spatial processing, and executive function. By calculating individual item scores, the scale allows for the detailed detection of impairment in one or more cognitive domains. Notably, the CFMSE not only addresses limitations in scoring found in older measurement tools but also features more comprehensive test items and a more detailed measurement process [5,6].

Diffusion Tensor Imaging (DTI) is widely used to detect microstructural damage in various brain diseases [7]. Derived from diffusion-weighted imaging techniques, DTI quantifies the Mean Diffusivity (MD) and Fractional Anisotropy (FA) of water molecules diffusing in the brain. MD reflects overall diffusivity (increased with tissue disruption), while FA reflects directional coherence of water diffusion (decreased with tract damage), thereby enabling the assessment of white matter fiber tract integrity [8,9].

The role of DTI in geriatric cognitive research is expanding [10]. Several studies have revealed a strong relationship between the integrity of white matter fiber tracts and cognitive impairment in older adults [11,12]. It has been argued that diffusion changes in key brain regions in MCI may occur earlier than changes in gray matter volume and may be more important for predicting MCI and AD [13]. For example, some studies have shown that FA values in whole-brain white matter tend to decrease linearly with age in older adults, particularly in the inferior frontal fasciculus, superior longitudinal fasciculus, inferior longitudinal fasciculus, and limbic pathways. This decrease in FA values is associated with a progressive loss of functional integrity [11,14]. In contrast, MD values in the hippocampus and temporoparietal white matter are elevated [15,16]. These findings reveal that changes in white matter fiber density and orientation, represented by MD and FA, play a key role in assessing brain microstructure.

Although most studies have confirmed that localized damage to white matter fiber tracts is associated with cognitive deficits in MCI, the specific areas of abnormality vary across studies [17,18,19]. In previous DTI studies, Region of Interest (ROI) and Voxel-Based Morphometry (VBM) have been the most frequently used analysis methods. ROI analysis is susceptible to researcher subjectivity, while VBM utilizes standard alignment algorithms and spatial smoothing processes that may affect result accuracy and tend to lead to false positives [20]. To address these limitations, Tract-Based Spatial Statistics (TBSS) has been developed. TBSS is a method based on the white matter skeleton that aims to compensate for bias caused by misalignment and smoothing kernel selection in voxel-based analysis, thus improving result accuracy and reducing false positive rates [21,22].

Despite the increasing application of TBSS to investigate white matter microstructural alterations in MCI using FA and MD metrics, most existing TBSS studies share two major limitations. First, they predominantly employ brief global cognitive screening tools (e.g., Mini-Mental State Examination, MMSE) or single-domain tests, which lack the granularity to capture the multidimensional nature of cognitive impairment in MCI. Consequently, the specific associations between white matter integrity in distinct tracts and domain-specific cognitive functions (e.g., memory, language, executive function) remain poorly understood. Second, the majority of these studies were conducted in Western clinical populations, leaving it unclear whether their findings generalize to community-dwelling older adults in non-Western cultural contexts, particularly China, where linguistic and educational backgrounds differ substantially.

To address these gaps, the present study makes three novel contributions. First, we employed the CFMSE, a comprehensive, culturally adapted instrument developed by Beijing Normal University based on the NIA-AA diagnostic criteria. Unlike brief screening tools, the CFMSE provides systematic, fine-grained domain-specific scores across five cognitive domains: memory, language, attention, spatial processing, and executive function. Second, we focused on a well-characterized Chinese community-dwelling elderly cohort, which enhances the generalizability of findings to non-Western, non-clinical aging populations. Third, we integrated CFMSE-derived domain-specific cognitive measures with TBSS-derived whole-brain white matter integrity indices (FA/MD). Accordingly, the primary objectives of this study are (1) to screen for the MCI cohort and characterize the pattern of cognitive domain impairments using the CFMSE; (2) to compare white matter microstructural integrity (FA/MD) between Chinese community-dwelling older adults with MCI and cognitively normal elderly using TBSS; and (3) to explore the domain-specific associations between cognitive function (measured by CFMSE subdomains) and white matter tract integrity (FA/MD), thereby providing a more comprehensive interpretation of the neural substrates underlying cognitive decline in MCI.

## 2. Methods

### 2.1. Data Acquisition

#### 2.1.1. Participant Recruitment and the Initial Screening

Elderly participants were recruited from several administrative districts in Shenzhen, specifically Nanshan, Bao’an, Longhua, and Guangming Districts. The inclusion criteria were as follows: age ≥ 60 years; normal or corrected-to-normal vision; right-handedness; being a native Chinese speaker; absence of organic brain disease, mental disorders, or major malignant diseases; and the ability to perform activities of daily living independently.

During the initial screening phase, basic demographic information was collected, including age, gender, education level, marital status, and the presence of chronic diseases (e.g., diabetes mellitus and hypertension). Screening also assessed for the presence of metal implants to ensure MRI compatibility. Subsequently, the MMSE was administered to assess global cognitive function; participants scoring ≥24 were eligible for inclusion. Finally, exclusion criteria included a lack of independent daily living abilities or the presence of severe comorbidities, as assessed by the Major Disease Exclusion Scale and the Activities of Daily Living (ADL) scale.

#### 2.1.2. The Secondary Screening: The Set of Cognitive–Behavioral Tests

The primary objective of the second screening stage was to comprehensively assess cognitive function in older adults who had passed the initial screening. Based on the assessment results, participants were classified into two groups: a cognitively normal group and an MCI group. The assessment protocol utilized the CFMSE developed by BNU, which comprises the following eight subtests: the Auditory Verbal Learning Test (AVLT), Complex Figure Test (CFT), Verbal Fluency Test (VFT), Boston Naming Test (BNT), Stroop Color Word Test (CWT), Trail Making Test (TMT), Clock Drawing Task (CDT), and the Symbol Digit Modalities Test (SDMT).

#### 2.1.3. Diagnostic Criteria

This study adopted the classification criteria proposed by Albert et al. (2011) [4] and Petersen and Morris (2005) [2], which are consistent with the diagnostic criteria for preclinical Alzheimer’s disease established by the NIA-AA. The diagnosis and cognitive function assessment of older adults with MCI were conducted based on the NIA-AA diagnostic criteria and the BNU version of the CFMSE. The specific diagnostic criteria for MCI were defined as follows: impairment in a cognitive domain was identified if two or more test scores within that domain fell below the cutoff (i.e., for a domain comprising two tests, both must be failed; for a domain comprising three tests, at least two must be failed). The presence of impairment in any of the five cognitive domains—namely memory, language, attention, spatial processing, and executive functioning—was considered indicative of MCI (see Table 1 for MCI Cognitive Performance Classification Criteria).

#### 2.1.4. DTI Data Acquisition

A 3T Siemens magnetic resonance scanner equipped with a high-speed gradient was used for acquisition, and diffusion-weighted imaging covering the whole brain was obtained by scanning parallel to the anterior and posterior commissure of the brain. The DTI parameters adopted in this study were as follows: Repetition Time (TR) = 3400 ms, Echo Time (TE) = 77.0 ms, Voxel Size = 1.6 × 1.6 × 1.6 mm, Slice thickness = 1.6 mm. FOV = 192 × 192 × 148 mm. b-value = 1000 s/mm^2^. In addition, we also performed reverse scanning. Detailed parameter settings are available in the Supplementary Materials.

### 2.2. Data Analysis

#### 2.2.1. Cognitive Test Scores

Demographic characteristics and MCI detection rates were calculated for both the normal control and MCI groups. Group differences in demographic variables, including gender, age, and education level, were subsequently analyzed. Cognitive performance scores were compared between the normal and MCI groups using independent samples *t*-tests, with statistical significance set at *p* < 0.05, effect sizes were calculated using Cohen’s d based on the pooled standard deviation. Pearson correlation analyses were conducted to examine the relationship between cognitive test scores and age within each group. To mitigate the probability of Type I errors, the Bonferroni correction was applied. All statistical analyses were performed using IBM SPSS Statistics 19.

#### 2.2.2. DTI Data Analysis

##### Data Preprocessing Based on TBSS

DTI data were primarily preprocessed using the Functional MRI Software Library (FSL, version 6.0). Tensor fitting was performed within the framework of the TBSS pipeline, utilizing the gradient information acquired during image acquisition. The specific processing steps were as follows:

(1) Data Quality Inspection: The number of gradient directions and b-values was verified, and the signal-to-noise ratio and artifacts were visually inspected to ensure data quality.
(2) Format Conversion: The original b0 images and 32 diffusion-weighted 2D images were converted into NIfTI format using the dcm2nii toolkit within MRIcron.
(3) Susceptibility and Eddy Current Correction: The TOPUP tool was employed to correct susceptibility-induced distortions by combining data from opposing phase-encode scans. Subsequently, Eddy Current Correction was applied to compensate for deformations arising from eddy currents and subject head motion during the scan.
(4) Gradient Direction Correction: The fdt_rotate_bvecs command was used to reorient the gradient vectors based on the spatial transformation parameters derived from the eddy current correction.
(5) Brain Extraction: The Brain Extraction Tool (BET) in FSL was utilized to extract brain tissue from the b0 images and generate brain masks, thereby improving the accuracy of subsequent spatial registration.
(6) Diffusion Tensor Calculation: Diffusion tensor models were fitted using the dtifit tool in FDT. This process generated diffusion tensors and calculated parametric maps for FA and MD.

##### FA

**Figure 1:**
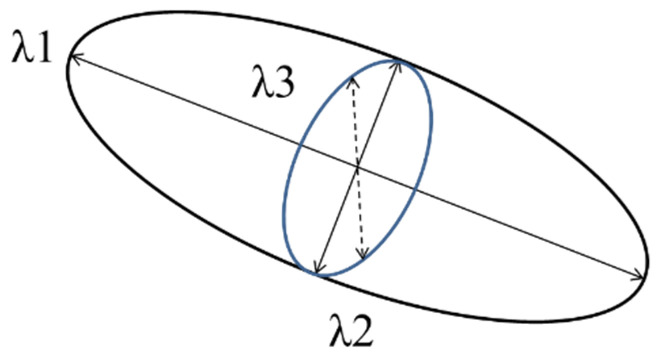
Schematic diagram of the FA calculation formula.

(1) Skeletonization: The TBSS pipeline was utilized to generate the mean FA skeleton. A fractional anisotropy threshold of 0.2 was applied to define the white matter skeleton [23], ensuring the exclusion of peripheral tracts with high inter-subject variability. The formula is (see Figure 1)

$$FA=\sqrt{\frac{3}{2}}\sqrt{\frac{{\left(\lambda 1-\lambda \right)}^{2}+{\left(\lambda 2-\lambda \right)}^{2}+{\left(\lambda 3-\lambda \right)}^{2}}{\left({\lambda 1}^{2}+{\lambda 2}^{2}+{\lambda 3}^{2}\right)}}\;\lambda =(\lambda 1+\lambda 2+\lambda )/3$$

(2) Design Matrix Generation: A design matrix for the two-sample *t*-test was constructed using the General Linear Model (GLM) toolkit, incorporating age and gender as covariates.
(3) Permutation Testing: Statistical inference was performed using the randomize command in FSL. A non-parametric permutation test was conducted on each voxel of the skeletonized data with 5000 permutations.
(4) Multiple Comparison Correction: Threshold-Free Cluster Enhancement (TFCE) was employed to correct for multiple comparisons (*p* < 0.05, statistically significant). TFCE was selected for its ability to avoid arbitrary cluster-forming thresholds, thereby improving statistical sensitivity and robustness while facilitating multi-scale signal detection. Regions exhibiting significant between-group differences were thereby identified.
(5) Cluster Visualization: The cluster command was used to identify significant clusters. Anatomical localization and visualization were performed using the “JHU White-Matter Tractography Atlas.”
(6) Correlation Analysis: Pearson correlation analysis, with Bonferroni correction applied to control for Type I errors, was conducted to explore the relationship between regional white matter alterations and cognitive test scores. Statistical significance was defined as *p* < 0.05.

##### MD

In a manner analogous to the FA analysis, MD maps were analyzed using the FSL software package. Statistical modeling was performed within the framework of the GLM to generate a design matrix for a two-sample *t*-test. Subsequently, non-parametric permutation testing (5000 permutations) was conducted on a voxel-wise basis using the randomize command. TFCE was applied to correct for multiple comparisons, with statistical significance defined as *p* < 0.05. Finally, the resulting statistical maps were visualized and anatomically labeled using the JHU white-matter tractography atlas. The formula is

$$MD=\frac{Tr\;(D)}{3}=\frac{D_{xx}+D_{yy}+D_{zz}}{3}$$

$$MD=\frac{trace(\overset{̿}{D})}{3}=\frac{\left(\lambda_{1}+\lambda_{2}+\lambda_{3}\right)}{3}$$

To further investigate the relationship between structural alterations and cognitive performance, Pearson correlation analyses were conducted on brain regions exhibiting significant between-group differences. The Bonferroni correction was applied to control for multiple comparisons, with statistical significance defined as *p* < 0.05.

## 3. Results

### 3.1. MCI Detection Rates and Demographic Information for Older Adults

A total of 130 elderly participants from Shenzhen were enrolled in this study, comprising 62 males (47.69%) and 68 females (52.31%). The cohort had a mean age of 66.0 ± 4.0 years and a mean educational level of 10.0 ± 2.9 years. Among them, 28 individuals (21.54%) were diagnosed with MCI, including 11 males (8.46%) and 17 females (13.08%) (see Table 2).

The independent samples *t*-test for demographic variables in the MCI and the cognitively normal elderly showed that the difference in age between the two groups was not significant (*t_age_* = −1.009, *p* = 0.315), and the difference in level of education was significant (*t_education_* = 3.833, *p* < 0.001), which means that education was a protective factor for MCI. The results of the chi-square analysis showed that there was no significant difference between the two groups in terms of gender (*χ*^2^*_gender_* = 1.011, *p* = 0.315), smoking (*χ*^2^*_smoking_* = 2.005, *p* = 0.157), chronic disease (*χ*^2^*_chronic disease_* = 0.134, *p* = 0.715), and marital status factors (*χ*^2^*_Marital status_* = 0.129, *p* = 0.719). Fisher’s exact test revealed a significant difference in alcohol use between the two groups (*p* = 0.045) (see Table 2).

### 3.2. The Cognitive–Behavioral Tests

#### 3.2.1. Comparison of Cognitive Performance Between MCI and Cognitively Normal Elderly

Independent samples *t*-tests showed that MCI had significantly lower MMSE scores than cognitively normal elderly (*t_MMSE_* = 3.926, *p* < 0.001). In the domain of language (*t_BOSTON_* = 9.912, *p* < 0.001; *t_VFT_* = 4.647, *p* < 0.001), attention (*t_SDMT_* = 6.291, *p* < 0.001; *t_TMT-A_* = −4.522, *p* < 0.001), memory (*t_REY-O Delay_* = 3.674, *p* < 0.001; *t_AVLT-N5_* = 6.797, *p* < 0.001; *t_AVLT-Total_* = 7.517, *p* < 0.001), executive function (*t_STROOP-C Time_* = −2.543, *p* = 0.016; *t_STROOP-C Number_* = 4.248, *p* < 0.001; *t_TMT-B_* = −5.027, *p* < 0.001) and spatial processing domain (*t_REY-O Copy_* = 4.457, *p* < 0.001; *t_CDT_* = 4.238, *p* < 0.001), MCI had significantly lower scores than cognitively normal elderly on all tests. It could be concluded that the overall cognitive function of MCI older adults in this study was significantly lower than that of normal older adults (see Table 3).

#### 3.2.2. Correlation Analysis of Age and Cognitive Scores Between MCI and Cognitively Normal Elderly

Pearson correlation analyses were conducted to examine the relationship between age and cognitive test scores within each group, treating age as a continuous variable. A Bonferroni correction was applied to control for multiple comparisons (corrected *α* = 0.05/13 ≈ 0.004). In the cognitively normal elderly group, age was significantly correlated with STROOP-C completion time (*r* = 0.377, *p* < 0.001) after correction, indicating an age-related decline in executive function. In the MCI group, the correlations between age and AVLT-Total score (*r* = −0.390, *p* = 0.040), as well as between age and TMT-B (*r* = 0.251, *p* = 0.011), did not remain significant after Bonferroni correction (see Table 4). The lack of significant associations with executive function tests in this population may reflect a floor effect; specifically, STROOP-C and TMT-B may have already been sufficiently challenging, thereby limiting the sensitivity to detect further age-related declines. Detailed comparison with age- and education-stratified normative data revealed that MCI participants performed 0.7–1.1 SD worse than healthy peers on the Stroop-C task [24]. More importantly, the variance in Stroop-C scores among MCI participants was significantly compressed at the slower end of the distribution (Levene’s test for equality of variances, *p* < 0.01), indicating a truncation of the score range. This floor effect obscures any potential linear relationship between age and executive function, as the task has become too challenging for the majority of MCI patients, regardless of their chronological age. This interpretation is further corroborated by the TMT-B data, where 32% of MCI participants failed to complete the test within the standard time limit.

### 3.3. Selection of Participants in the DTI Experiment

**Figure 2:**
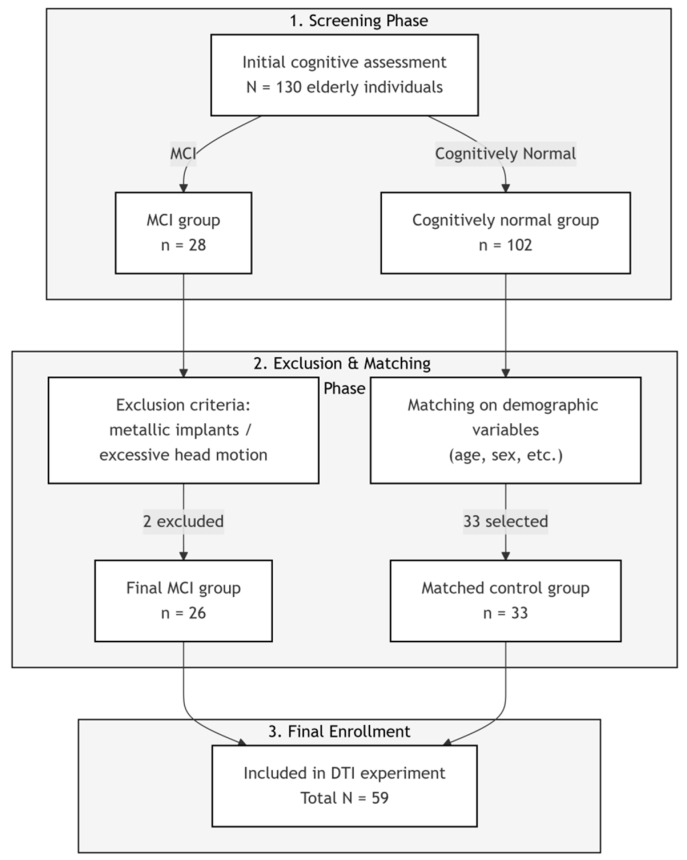
Study flow diagram of participant selection for the DTI experiment.

From an initial cohort of 130 older adults who underwent cognitive screening, 59 participants were recruited for the DTI study. The MCI group included 26 individuals selected from 28 eligible candidates; two were excluded due to MRI contraindications (metallic implants) or excessive head motion artifacts (FD > 0.5 mm). To ensure group comparability, 33 cognitively normal older adults were selected as healthy controls and matched to the MCI group on age and sex. The final sample comprised 26 individuals with MCI (9 males, 17 females; mean age: 67.3 ± 4.8 years) and 33 healthy controls (12 males, 21 females; mean age: 65.8 ± 4.5 years). The participant recruitment and screening process is detailed in Figure 2. Detailed demographic characteristics and neuropsychological profiles for both groups are summarized in Table 5 and Table 6.

### 3.4. DTI

#### 3.4.1. Comparison of FA Between MCI and Cognitively Normal Elderly

**Figure 3:**
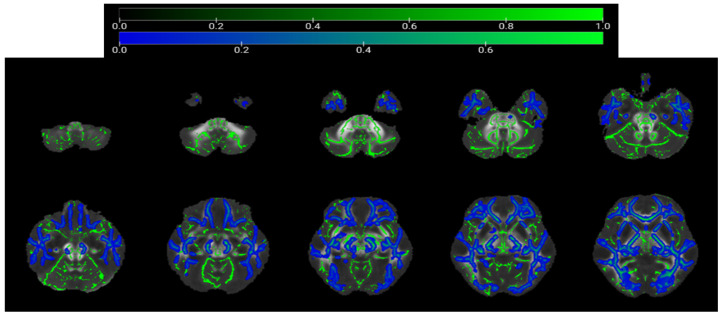
Comparison of FA values between MCI and cognitively normal elderly. Note: In the figure, the white areas represent the white matter skeleton, while the blue-green regions indicate areas with reduced FA values in the MCI group compared to the cognitively normal elderly. Specifically, regions reaching statistical significance are highlighted in blue. Table 7 provides detailed information for each region exhibiting significant differences shown in the figure.

Group differences were assessed using a GLM, with age and gender included as nuisance covariates. Analysis based on the JHU template revealed that the MCI group exhibited significantly reduced FA values in several white matter tracts compared to the cognitively normal elderly group (TFCE-corrected *p* < 0.05). These regions included the corpus callosum, internal capsule, corona radiata, thalamic radiation, external capsule, superior fronto-occipital fasciculus, and cingulum (cingulate gyrus). No clusters of significantly increased FA values were observed in the MCI group. Figure 3 illustrates the spatial distribution of significant FA differences between the groups (TFCE-corrected), while Table 7 details the specific regions and their corresponding T-values based on the JHU atlas.

#### 3.4.2. Comparison of MD Between MCI and Cognitively Normal Elderly

**Figure 4:**
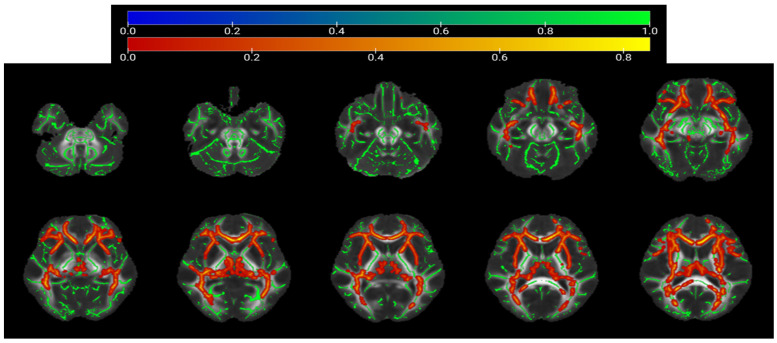
Comparison of MD values between MCI and cognitively normal elderly. Note: In the figure, the white areas represent the white matter skeleton, while the yellow–red regions indicate areas with increased MD values in the MCI group compared to the cognitively normal elderly. Specifically, regions reaching statistical significance are highlighted in red. Table 8 provides detailed information for each region exhibiting significant differences shown in the figure.

**Figure 5:**
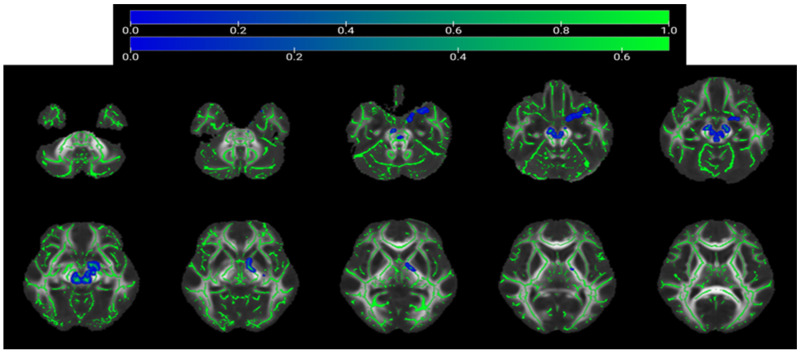
Comparison of MD values between MCI and cognitively normal elderly. Note: In the figure, the white areas represent the white matter skeleton, while the blue-green regions indicate areas with reduced MD values in the MCI group compared to the cognitively normal elderly. Specifically, regions reaching statistical significance are highlighted in blue. Table 9 provides detailed information for each region exhibiting significant differences shown in the figure.

Group differences were assessed using a General Linear Model (GLM), with age and gender included as nuisance covariates. Analysis based on the JHU template revealed that the MCI group exhibited significantly increased MD values in the corpus callosum, corona radiata, thalamic radiation, internal capsule, external capsule, superior fronto-occipital fasciculus, and cingulum (cingulate gyrus) compared to the cognitively normal elderly group (TFCE-corrected *p* < 0.05). Conversely, significantly decreased MD values were observed in the MCI group within the cingulum (hippocampus), inferior cerebellar peduncle, and corticospinal tract (TFCE-corrected *p* < 0.05). Figure 4 and Figure 5 illustrate the spatial distribution of significant MD differences between the groups (TFCE-corrected), while Table 8 and Table 9 detail the specific regions and their corresponding T-values based on the JHU atlas.

### 3.5. Correlation Analysis Between DTI Data and Cognitive Test Scores in the MCI

#### 3.5.1. Correlation Analysis Between FA of White Matter Structure and Cognitive Test Scores

**Figure 6:**
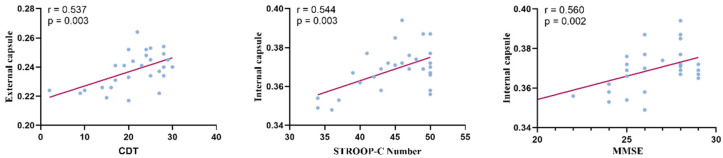
Correlation between FA values and cognitive test scores in MCI. Note: The figure above illustrates the correlations between FA values of white matter fiber tracts and cognitive test scores in regions exhibiting significant differences between the MCI and normal groups. Specifically, after Bonferroni correction, the positive correlations remained significant (*p* < 0.004) between the posterior limb of the internal capsule and MMSE/STROOP-C scores, and between the external capsule and CDT scores.

To examine the relationship between regional FA values and cognitive performance, Pearson correlations were conducted separately for each brain region that showed significant group differences. For each region, 13 correlations were performed (one for each cognitive test score). To control for the family-wise error rate within each region, a region-wise Bonferroni correction was applied, with the significance threshold set at corrected α = 0.05/13 ≈ 0.004. The results indicated that FA values in the posterior limb of the internal capsule and the external capsule were significantly and positively correlated with scores on several cognitive tests, as shown in Figure 6 and Table 10.

#### 3.5.2. Correlation Analysis Between MD of White Matter Structure and Cognitive Test Scores

**Figure 7:**
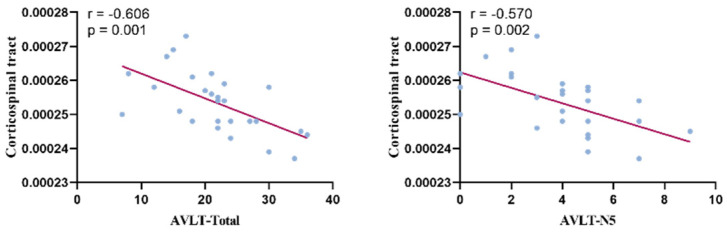
Correlation between MD values and cognitive test scores in MCI. Note: The figure above illustrates the correlations between MD values of white matter fiber tracts and cognitive test scores in regions exhibiting significant differences between the MCI and normal groups. Specifically, after Bonferroni correction, the negative correlations remained significant (*p* < 0.004) between the orticospinal tract and AVLT-N5/AVLT-Total scores.

To examine the relationship between regional MD values and cognitive performance, Pearson correlations were conducted separately for each brain region that showed significant group differences. The results revealed that MD values in the corticospinal tract were significantly negatively correlated with scores on several cognitive tests, as depicted in Figure 7 and Table 11.

## 4. Discussion

### 4.1. MCI Detection Rates and Characteristics of Changes in Cognitive Functions

Accurate and efficient identification of patients with MCI among the cognitively normal elderly population is a prerequisite for both basic and applied research on MCI, as well as a critical component of early dementia prevention and intervention. In the present study, 130 older adults from various districts of Shenzhen were assessed, and 28 individuals with MCI were identified, yielding a detection rate of 21.54%. This result aligns with the overall MCI prevalence reported in Jia et al.’s (2020) [1] study on cognitive impairment among Chinese older adults, as well as with the 15.4% to 32% range documented in recent studies on community-based MCI screening and diagnostic management [1,25]. With the accelerating trend of population aging, the prevalence of MCI among individuals aged 60 years and above in China has been steadily increasing. Notably, the prevalence in the 80- to 90-year-old age group is 8 to 15 percentage points higher than that in the 70- to 80-year-old and 60- to 70-year-old groups [26]. Most participants in the present study were in the 60- to 70-year-old age range, a group characterized by relatively stable cognitive functioning; consequently, the observed MCI prevalence was not significantly elevated. Furthermore, previous studies have indicated that the source of participants may influence MCI detection rates [27,28], though findings on this topic remain inconsistent. For instance, some studies have reported a high MCI prevalence of up to 85% in clinical outpatient settings, whereas community-based screening has yielded a lower prevalence of approximately 23.4% [29]. A meta-analysis comparing outpatient and community detection rates found an overall MCI prevalence of 21% (95% CI = 17–-26%), with community populations showing a slightly higher prevalence (25%) than clinical outpatient groups (20%) [30]. Given that the older adults in the present study were recruited from various communities in Shenzhen, the observed MCI detection rate falls within the expected and reasonable range.

MCI is regarded as the “window period” for AD, characterized primarily by significant memory decline [2]. In the present study, scores across various cognitive domains were significantly lower in the MCI group compared to the cognitively normal elderly group. Previous studies have shown that MCI is not limited to isolated memory impairment but may also involve functional deficits in multiple cognitive domains, such as language, attention, and executive function [5,31]. Compared with amnestic MCI, which is primarily characterized by memory impairment, MCI involving multiple cognitive domain deficits presents with more severe symptoms, poorer clinical treatment outcomes, and a higher likelihood of progression to AD. Furthermore, Pearson correlation analyses of cognitive function with age in both MCI and cognitively normal elderly groups revealed a significant age-related decline in memory function among MCI patients. However, while cognitive behavioral measures can identify phenotypic changes in cognitive function, the underlying neural mechanisms remain complex. Therefore, we employed DTI to further explore the neural mechanisms associated with MCI.

### 4.2. Fractional Anisotropy Changes in White Matter Fibers in MCI

Cerebral white matter fiber tract FA values reflect the degree of anisotropy in water molecule diffusion, with values ranging from 0 to 1. The closer the FA value is to 1, the greater the anisotropy of water molecule diffusion along the nerve fiber tracts. Higher FA values typically indicate that water molecules diffuse primarily parallel to the white matter fiber tracts; this phenomenon is often interpreted as reflecting more organized, healthy, or mature white matter [32]. In contrast, the closer the FA value is to 0, the more the diffusive movement tends toward isotropy. For instance, when nerve fiber tracts are compromised, water molecules exhibit greater diffusion in other directions, resulting in a lower FA value.

In the present study, we found that compared to cognitively normal elderly individuals, patients with MCI had significantly decreased FA values in the genu and body of the corpus callosum (association fibers). This result was consistent with structural imaging findings in previous studies [33]. A study by Seiler (2018) also indicated that the body of the corpus callosum is one of the most vulnerable regions in terms of white matter microstructure [34]. In addition, the present study found that the FA values of the corona radiata, internal capsule, external capsule, and posterior thalamic radiations (projection fibers) in the MCI group showed a significant decrease. The white matter fiber tracts in these regions are responsible for information transmission and coordination, which are important for sensory and visual information processing. The results of the correlation analysis in the present study also showed that the FA values of the internal capsule and external capsule were significantly and positively correlated with scores on several cognitive tests in the domains of spatial information processing and executive function. This suggests that the integrity of white matter fiber tracts in the frontal-parietal–temporal regions of MCI patients was reduced, and white matter microstructure was impaired. This might be due to axonal damage and myelin loss in the frontal-parietal–temporal region, which are most likely to occur in the brains of older adults with MCI [33,35].

Finally, we found that the FA values of the superior fronto-occipital fasciculus and cingulum (cingulate gyrus) in the MCI group were significantly reduced. Previous studies have reported that FA values in the hippocampus, posterior limb of the internal capsule, corpus callosum, as well as the superior and inferior longitudinal fasciculus, temporal lobe white matter, and parietal lobe white matter, were significantly lower in older adults with MCI than in cognitively normal elderly individuals [15,16]. This finding suggests that older adults with MCI may exhibit a broader range of alterations in FA values across white matter fiber tracts.

### 4.3. Change in Diffusivity of White Matter Fibers in MCI

The MD value of white matter fiber tracts reflects the overall diffusion level of water molecules, independent of diffusion direction. While FA is highly sensitive to changes in fiber orientation, MD is more sensitive to changes in fiber density [36]. In DTI studies, a decrease in FA value and an increase in MD value reflect enhanced diffusion of water molecules within white matter fiber tracts, which may indicate damage to the integrity of the myelin sheath [37,38]. The present study revealed that MD values in the corpus callosum, corona radiata, thalamic radiation, internal capsule, external capsule, superior fronto-occipital fasciculus, and cingulum (cingulate gyrus) were significantly increased in the MCI group. Furthermore, MD values of the corticospinal tract showed a significant negative correlation with memory test scores. This result was consistent with the FA findings, indicating that patients with MCI had white matter microstructural damage in the aforementioned brain regions; this conclusion is also supported to some extent by previous research [39,40]. Furthermore, these results indicate that FA and MD values play a significant role in cognition, a viewpoint consistent with previous research findings [41,42].

In the aforementioned brain regions, the increased MD values of white matter fiber tracts in the MCI group indicated a higher degree of free water diffusion within the tracts. This suggests that white matter degeneration in MCI begins with demyelination of fiber tracts or secondary axonal degeneration, ultimately disrupting the structural integrity of nerve fiber tracts [43]. In addition, other studies have found that elderly individuals with MCI exhibit damage to both the internal and external central cholinergic projection pathways, manifested by decreased FA values in the cingulate gyrus and increased MD values in the external capsule [44]. These results are consistent with the findings of the present study. Regarding this phenomenon, researchers have speculated that these pathways traverse areas prone to cerebrovascular disease, making them more susceptible to vascular damage and leading to early cognitive impairment [45].

Unlike previous studies that have reported significantly higher MD values in the cingulum (hippocampus) in MCI and AD patients [46], the present study found significantly lower MD values in this region, as well as in the inferior cerebellar peduncle and corticospinal tract, in older adults with MCI. The cingulum (hippocampus) is a cortico-limbic tract that originates from the precuneus region of the hippocampus and intersects with the cingulate gyrus; it serves as a key relay region of the cortico-limbic system and plays a critical role in cognitive functions such as memory [47]. In the present study, this region did not show significant alterations in FA analyses, yet its MD value was significantly lower.

Several explanations—both biological and technical—may account for this unexpected finding, although caution is warranted given the divergence from the existing literature. A longitudinal study reported that while most regions of the aging brain exhibit a trend of gradually decreasing FA and increasing MD, some brain regions show the opposite trend [42]. Biologically, reactive gliosis or fiber reorganization could, in theory, give rise to decreased MD and increased FA in specific regions [42,48,49]. However, far more parsimonious technical explanations must be considered before such biological interpretations can be substantiated. First, partial volume effects from adjacent cerebrospinal fluid (CSF) and age-related atrophy—particularly in thin tracts such as the hippocampal cingulum—can bias MD estimates downward [50,51,52]. Second, the hippocampal cingulum is particularly susceptible to TBSS skeletonization errors, as the population-mean skeleton often fails to accurately track individual anatomy in this region [53]. While extracting a ‘white matter skeleton’ helps reduce registration errors, it represents only a population-averaged centerline. This limitation can result in the loss of critical white matter information, potentially introducing false-positive or false-negative results [54]. Third, free-water contamination in atrophied tissue can produce asymmetric and potentially confounding effects on diffusion metrics [55]. In addition, the specific scanning protocol (e.g., repetition time, number of signal averages) may also influence MD estimates, with longer repetition times and increased signal averaging leading to lower MD values [56]. Given the modest sample size of the present study, the possibility that outliers disproportionately contributed to the observed MD decrease cannot be excluded.

Regarding the comparison between MD and FA, some studies have suggested that MD may capture more widespread alterations in white matter integrity than FA in MCI, potentially reflecting subtle microstructural damage not yet detectable by FA [57,58]. A longitudinal study reported that while the demyelination index increases in normal-appearing white matter (NAWM), and FA changes in normal white matter are associated with overall cognitive performance, such changes are not observed in white matter hyperintensities (WMH) [59]. Research comparing WMH and normal white matter integrity in the aging brain has shown that MD provides the clearest distinction between NAWM and WMH [57]. Pathological studies indicate that WMH corresponds to tissue changes such as edema, demyelination, and axonal loss, which often occur at a later stage [60]. With advancing age, the blood–brain barrier gradually deteriorates [61], leading to increased interstitial fluid and perivascular tissue damage; these processes may also be reflected in changes to imaging markers [60]. However, it is only when WMH becomes more severe that FA begins to reflect significant changes in tissue microstructure [57]. Nevertheless, caution is warranted when interpreting these findings, as several methodological limitations must be acknowledged. Notably, when using FA and MD as indicators of white matter integrity, these metrics cannot fully isolate the contribution of individual fibers within voxels characterized by complex multi-fiber architectures, such as crossing fibers [62,63,64].

Collectively, our findings highlight the relevance of white matter integrity in MCI [65,66]. Furthermore, this study reinforces the potential utility of assessing diffusion biomarkers within critical neural pathways, such as the cingulum (hippocampus) [13].

### 4.4. Limitations and Perspectives

The findings of this study should be interpreted in light of several limitations. First, the relatively small sample size warrants caution regarding the reliability and reproducibility of the results. Due to sample size constraints, all MCI subtypes were collapsed into a single group. Given that amnestic and non-amnestic MCI are known to exhibit distinct white matter profiles [18], the heterogeneity of our MCI cohort represents a potential confounding factor. Furthermore, the cross-sectional design precludes the determination of developmental trends, stability, or trajectories of white matter changes in individuals with MCI. Future large-scale, longitudinal studies are warranted to enhance the ecological validity of these findings and to further clarify the relationship between white matter alterations and cognitive performance in MCI. This represents another important direction for future research.

Second, the etiology and population characteristics of MCI are multidimensional and complex. There were significant differences in education levels between the MCI and HC groups. Although education can influence cognitive reserve and brain structure, perfect matching was constrained by the clinical characteristics of the patient population. While our findings primarily highlight disease-specific white matter alterations, the potential confounding effect of education should be considered, and future studies with strictly matched cohorts are warranted.

Third, the present study primarily focused on FA and MD metrics, while axial and radial diffusivity were not examined; these parameters warrant further exploration in subsequent studies. Additionally, it is estimated that 60% to 90% of voxels within the white matter contain crossing fibers [67]. Future research could employ the “fixel” metric to calculate the fiber orientation distribution (FOD) of the fiber bundle within each voxel using constrained spherical deconvolution [68,69]. Although structural changes in the brain can indirectly reflect the state of cognitive function, they do not fully predict it. Therefore, the specific correlation between white matter fiber tract integrity and cognitive decline in MCI requires further investigation.

Finally, this study utilized DTI combined with TBSS to analyze white matter microstructure damage in older adults with MCI. Compared to standard TBSS, the framework proposed by TBSS++ exhibits significantly improved reproducibility and robustness [54].

## 5. Conclusions

The detection rate of MCI in the present study was approximately 21.54%. Elderly individuals with MCI exhibited significant declines in memory, attention, language, executive function, and spatial processing, accompanied by widespread damage to white matter microstructure. Furthermore, these observations tentatively suggest that alterations in white matter fiber density might be associated with microstructural changes in the brains of MCI patients. This could potentially underscore the value of exploring diffusion biomarkers in key regions such as the cingulum (hippocampus).

## Figures and Tables

**Table 1:** MCI Cognitive Performance Classification Criteria.

Index Age Group	$\bar{x}$-1.5 SD	
	60–69	70–79
Memory		
AVLT-N5	3	2
AVLT-Total	18	15
REY-O Delay	8	6
Spatial Processing		
REY-O Copy	30	29
CDT	17	
Language		
VFT	10	
BNT	20	
Attention		
SDMT	29	
TMT-A Middle school ^#^	90	120
TMT-A College ^#^	80	85
Executive Function		
Stroop-C Time	111	130
Stroop-C Number	38	35
TMT-B Middle school ^#^	230	290
TMT-B College ^#^	210	240

Notes: ^#^ Education: Middle school = (6, 12]; College ≥ 13.

**Table 2:** Demographic information of the MCI and cognitively normal elderly groups.

Item	Cognitively Normal Elderly (N = 102)	MCI (N = 28)	Difference Examination		
*Continuous variable*			*t*	*p*	*df*
Age	66.2 ± 4.5	67.1 ± 4.7	−1.009	0.315	128
Education (year)	10.4 ± 2.8	8.1 ± 2.9	3.833	<0.001	128
*Categorical variable*			*χ* ^2^	*p*	*df*
Gender			1.011	0.315	1
Male	51 (50.00%)	11 (39.29%)			
Female	51 (50.00%)	17 (60.71%)			
Alcohol addiction				0.045 ^#^	
Yes	0 (0%)	2 (7.14%)			
No	102(100%)	26 (92.86%)			
Smoking			2.005	0.157	1
Yes	17 (16.67%)	8 (28.57%)			
No	85 (83.33%)	20 (71.43%)			
Chronic disease			0.134	0.715	1
Yes	44 (43.14%)	11 (39.29%)			
No	58 (56.86%)	17 (60.71%)			
Marital status			0.129	0.719	1
Married	90 (88.24%)	24 (85.71%)			
Widow/Divorce	12 (11.76%)	4 (14.29%)			

Note: ^#^ Fisher’s exact test was employed due to the absence of participants reporting alcohol use in the cognitively normal elderly group.

**Table 3:** Independent sample *t*-test of cognitive scores between MCI and cognitively normal elderly.

Domain	Test	Cognitively Normal Elderly (N = 102)	MCI (N = 28)	*t*	*95% CI*	*Effect Size*
	MMSE	26.57 ± 1.59	25.00 ± 2.68	3.926 ***	0.778–2.359	0.84
**Language**	BOSTON	25.02 ± 3.09	19.54 ± 6.65	9.912 ***	5.317–7.969	1.34
	VFT	18.89 ± 4.17	14.68 ± 4.52	4.647 ***	2.419–6.008	1.00
**Attention**	SDMT	33.92 ± 8.71	22.96 ± 8.01	6.291 ***	7.451–14.463	1.29
	TMT-A	54.04 ± 13.34	82.99 ± 33.15	−4.522 ***	−42.039–−15.867	1.51
**Memory**	REY-O Delay	14.54 ± 6.65	9.54 ± 5.30	3.674 ***	2.309–7.698	0.79
	AVLT-N5	6.05 ± 2.10	2.93 ± 2.32	6.797 ***	2.212–4.029	1.46
	AVLT-Total	29.52 ± 7.39	17.82 ± 6.91	7.517 ***	8.619–14.777	1.62
**Executive Function**	STROOP-C Time	82.49 ± 19.51	97.27 ± 29.01	−2.543 *	−26.59–−2.970	0.68
	STROOP-C Number	47.41 ± 3.19	43.11 ± 5.09	4.248 ***	2.243–6.366	1.18
	TMT-B	154.25 ± 48.84	250.87 ± 98.42	−5.027 ***	−135.830–−57.409	1.55
**Spatial Processing**	REY-O Copy	33.25 ± 2.68	26.93 ± 7.38	4.457 ***	3.423–9.229	1.54
	CDT	24.50 ± 2.98	17.86 ± 3.67	4.238 ***	2.842–8.125	2.13

Note: * *p* < 0.05, *** *p* < 0.001.

**Table 4:** Pearson’s correlation analysis of cognitive scores with age in MCI and cognitively normal elderly.

Domain	Test	Cognitively Normal Elderly (N = 102)		MCI (N = 28)	
		*r*	*p*	*r*	*p*
	MMSE	0.034	0.737	0.252	0.195
Language	BOSTON	0.118	0.236	−0.170	0.387
	VFT	0.073	0.467	−0.279	0.150
Attention	SDMT	−0.192	0.053	−0.085	0.666
	TMT-A	0.131	0.191	−0.034	0.863
Memory	REY-O Delay	−0.002	0.987	−0.211	0.282
	AVLT-N5	−0.103	0.301	−0.037	0.853
	AVLT-Total	−0.125	0.212	−0.390 *	0.040
Executive function	STROOP-C Time	0.377 ***	<0.001	0.218	0.266
	STROOP-C Number	−0.066	0.511	0.166	0.397
	TMT-B	0.251 *	0.011	0.103	0.601
Spatial Processing	REY-O Copy	−0.013	0.899	0.012	0.951
	CDT	−0.036	0.718	0.246	0.207

Note: * *p* < 0.05, *** *p* < 0.001.

**Table 5:** Demographic characteristics of participants in the DIT experiment.

Item	Cognitively Normal Elderly (N = 33)	MCI (N = 26)	Difference Examination		
*Continuous variable*			*t*	*p*	*df*
Age	65.8 ± 4.5	67.3 ± 4.8	−1.168	0.248	57
Education (year)	10.9 ± 2.3	8.3 ± 3.0	3.859	<0.001	57
*Categorical variable*			*χ* ^2^	*p*	*df*
Gender			0.019	0.889	1
Male	12 (36.36%)	9 (34.62%)			
Female	21 (63.64%)	17 (65.38%)			
Alcohol addiction				0.190 ^#^	
Yes	0 (0%)	2 (7.69%)			
No	33 (100%)	24 (92.31%)			
Smoking			3.285	0.070	1
Yes	3 (9.09%)	7 (26.92%)			
No	30 (90.91%)	19 (73.08%)			
Chronic disease			2.853	0.091	1
Yes	20 (60.61%)	10 (38.46%)			
No	13 (39.39%)	16 (61.54%)			
Marital status			2.862	0.091	1
Married	32 (96.97%)	22 (84.62%)			
Widow/Divorce	1 (3.03%)	4 (15.38%)			

Note: ^#^ Fisher’s exact test was employed due to the absence of participants reporting alcohol use in the cognitively normal elderly group.

**Table 6:** Independent sample *t*-test of cognitive scores between MCI and cognitively normal elderly in the DIT experiment.

Domain	Test	Cognitively Normal Elderly (N = 33)	MCI (N = 26)	*t*	*95% CI*	*Effect Size*
	MMSE	26.97 ± 1.99	25.00 ± 2.77	3.175 **	0.728–3.121	0.85
Language	BOSTON	24.03 ± 2.94	17.77 ± 3.63	7.325 ***	4.549–7.973	1.95
	VFT	18.76 ± 3.18	14.46 ± 4.11	4.527 ***	2.396–6.196	1.21
Attention	SDMT	31.09 ± 8.76	23.12 ± 8.28	3.555 **	3.483–12.468	0.95
	TMT-A	56.02 ± 14.79	82.75 ± 34.42	−3.700 **	−41.437–−12.018	1.07
Memory	REY-O Delay	13.52 ± 6.45	9.35 ± 5.39	2.646 *	1.014–7.324	0.71
	AVLT-N5	5.39 ± 2.02	2.81 ± 2.26	4.636 ***	1.469–3.703	1.23
	AVLT-Total	26.70 ± 7.65	17.69 ± 6.64	4.754 ***	5.211–12.798	1.27
Executive Function	STROOP-C Time	83.61 ± 14.57	96.47 ± 29.30	−2.047 *	−25.611–−0.102	0.59
	STROOP-C Number	47.61 ± 2.30	42.88 ± 5.22	4.296 ***	2.484–6.958	1.25
	TMT-B	161.28 ± 49.07	250.13 ± 98.70	−4.199 ***	−131.818–−45.883	1.21
Spatial Processing	REY-O Copy	33.00 ± 2.95	26.92 ± 7.23	4.029 ***	3.003–9.151	1.17
	CDT	25.79 ± 2.86	19.85 ± 6.81	4.169 ***	3.038–8.845	1.21

Note: * *p* < 0.05, ** *p* < 0.01, *** *p* < 0.001.

**Table 7:** The regions with significantly lower FA values in MCI (TFCE corrected).

Region	T-Value	*p*
Genu of corpus callosum	−5.15	<0.001
Body of corpus callosum	−5.59	<0.001
Anterior limb of internal capsule R	−2.46	0.017
Anterior limb of internal capsule L	−2.35	0.022
Posterior limb of internal capsule R	−3.40	0.001
Posterior limb of internal capsule L	−3.94	<0.001
Anterior corona radiata R	−3.26	0.002
Anterior corona radiata L	−3.93	<0.001
Superior corona radiata R	−5.86	<0.001
Superior corona radiata L	−6.08	<0.001
Posterior corona radiata R	−2.61	0.012
Posterior corona radiata L	−4.55	<0.001
Posterior thalamic radiation R	−2.75	0.008
External capsule R	−5.65	<0.001
External capsule L	−5.89	<0.001
Superior fronto-occipital fasciculus R	−2.05	0.045
Superior fronto-occipital fasciculus L	−2.08	0.042
Cingulum (cingulate gyrus) R	−2.56	0.013
Cingulum (cingulate gyrus) L	−2.88	0.006

Note: The regions listed above are white matter fiber tracts that remained significantly different after multiple corrections (JHU template), *p* < 0.05.

**Table 8:** The regions with significantly higher MD values in MCI (TFCE corrected).

Region	T-Value	*p*
Genu of corpus callosum	6.04	<0.001
Body of corpus callosum	4.72	<0.001
Anterior corona radiata R	4.74	<0.001
Anterior corona radiata L	4.48	<0.001
Superior corona radiata R	3.12	0.003
Superior corona radiata L	3.77	<0.001
Posterior corona radiata R	2.69	0.010
Posterior corona radiata L	3.19	0.002
Posterior thalamic radiation R	2.60	0.012
Posterior limb of internal capsule R	3.26	0.002
External capsule R	3.40	0.001
External capsule L	3.17	0.003
Superior fronto-occipital fasciculus R	2.97	0.004
Superior fronto-occipital fasciculus L	3.71	<0.001
Cingulum (cingulate gyrus) R	3.53	<0.001
Cingulum (cingulate gyrus) L	4.03	<0.001

Note: The regions listed above are white matter fiber tracts that remained significantly different after multiple corrections (JHU template), *p* < 0.05.

**Table 9:** The regions with significantly lower MD values in MCI (TFCE corrected).

Region	T-Value	*p*
Cingulum (hippocampus) R	−2.05	0.045
Cingulum (hippocampus) L	−2.23	0.030
Inferior cerebellar peduncle R	−4.41	<0.001
Inferior cerebellar peduncle L	−4.17	<0.001
Corticospinal tract R	−2.59	0.012
Corticospinal tract L	−3.66	<0.001

Note: The regions listed above are white matter fiber tracts that remained significantly different after multiple corrections (JHU template), *p* < 0.05.

**Table 10:** Correlation analysis between FA values and cognitive test scores in MCI (Bonferroni corrected).

Region	Test	*r*	*p*
Posterior limb of internal capsule	MMSE	0.560	0.002
	STROOP-C Number	0.544	0.003
External capsule	CDT	0.537	0.003

**Table 11:** Correlation analysis between MD values and cognitive test scores in MCI (Bonferroni corrected).

Region	Test	*r*	*p*
Corticospinal tract	AVLT-N5	−0.570	0.002
	AVLT-Total	−0.606	0.001

## Data Availability

The original contributions presented in this study are included in the article/Supplementary Materials. Further inquiries can be directed to the corresponding author.

## Supplementary Materials

The following supporting information can be downloaded at: https://www.mdpi.com/article/10.3390/brainsci16060655/s1. The supplementary materials include the participants’ informed consent forms and the parameters.
