# Supplementary material for: The Association Between Changes in White Matter Microstructure and Cognitive Function in Older Adults with Mild Cognitive Impairment
Source: Brain Sci. 2026 Jun 22;16(6):655. doi: 10.3390/brainsci16060655 (PMC13296742; doi:10.3390/brainsci16060655)
Supplement: Supplementary file 1 [file brainsci-16-00655-s001.zip › Parameters.pdf]

\\USER\CainAkatsuki\Language\L-CA\ep2d_diff

TA: 7:41 PM: REF Voxel size: 1.6×1.6×1.6 mmPAT: 4 Rel. SNR: 1.00 : epse

Properties

Prio recon	Off
Load images to viewer	On
Inline movie	Off
Auto store images	On
Load images to stamp segments	Off
Load images to graphic segments	Off
Auto open inline display	Off
Auto close inline display	Off
Start measurement without further preparation	Off
Wait for user to start	Off
Start measurements	Single measurement

Routine

Slice group	1
Slices	92
Dist. factor	0 %
Position	L0.0 A7.3 F3.5 mm
Orientation	T > C-5.7
Phase enc. dir.	A >> P
AutoAlign	Head > Brain
Phase oversampling	0 %
FoV read	192 mm
FoV phase	100.0 %
Slice thickness	1.6 mm
TR	3400 ms
TE	77.0 ms
Concatenations	1
Filter	None
Coil elements	HC1-7

Contrast - Common

TR	3400 ms
TE	77.0 ms
MTC	Off
Magn. preparation	None
Fat suppr.	Fat sat.
Fat sat. mode	Weak

Contrast - Dynamic

Averaging mode	Long term
Reconstruction	Magnitude
Measurements	1
Delay in TR	0 ms
Multiple series	Off

Resolution - Common

FoV read	192 mm
FoV phase	100.0 %
Slice thickness	1.6 mm
Base resolution	120
Phase resolution	100 %
Phase partial Fourier	6/8
Interpolation	Off

Resolution - iPAT

Accel. mode	Slice accel.
Accel. factor PE	1
Ref. lines PE	12
Accel. factor slice	4

Resolution - iPAT

Reference scan mode	EPI/separate

Resolution - Filter Image

Distortion Corr.	Off
Prescan Normalize	Off
Dynamic Field Corr.	Off

Resolution - Filter Rawdata

Raw filter	Off
Elliptical filter	Off

Geometry - Common

Slice group	1
Slices	92
Dist. factor	0 %
Position	L0.0 A7.3 F3.5 mm
Orientation	T > C-5.7
Phase enc. dir.	A >> P
FoV read	192 mm
FoV phase	100.0 %
Slice thickness	1.6 mm
TR	3400 ms
Multi-slice mode	Interleaved
Series	Interleaved
Concatenations	1

Geometry - AutoAlign

Slice group	1
Position	L0.0 A7.3 F3.5 mm
Orientation	T > C-5.7
Phase enc. dir.	A >> P
AutoAlign	Head > Brain
Initial Position	L0.0 A7.3 F3.5
R	0.0 mm
A	7.3 mm
F	3.5 mm
Initial Rotation	0.07 deg
Initial Orientation	T > C
T > C	-5.7
> S	0.0

Geometry - Saturation

Fat suppr.	Fat sat.
Fat sat. mode	Weak
Special sat.	None

Geometry - Navigator

System - Miscellaneous

Positioning mode	REF
Table position	H
Table position	0 mm
MSMA	S - C - T
Sagittal	R >> L
Coronal	A >> P
Transversal	F >> H
Coil Combine Mode	Adaptive Combine
Matrix Optimization	Performance
AutoAlign	Head > Brain
Coil Select Mode	Default

System - Adjustments

B0 Shim mode	Standard
B1 Shim mode	TrueForm
Adjust with body coil	On
Confirm freq. adjustment	Off
Assume Dominant Fat	Off
Assume Silicone	Off
Adjustment Tolerance	Auto

System - Adjust Volume

Position	L0.0 A7.3 F3.5 mm
Orientation	T > C-5.7
Rotation	0.07 deg
A >> P	192 mm
R >> L	192 mm
F >> H	148 mm
Reset	Off

System - pTx Volumes

B1 Shim mode	TrueForm
Excitation	Standard

System - Tx/Rx

Frequency 1H	123.262143 MHz
Correction factor	1
Gain	High
Img. Scale Cor.	1.000
Reset	Off
? Ref. amplitude 1H	0.000 V

Physio - Signal1

1st Signal/Mode	None
TR	3400 ms
Concatenations	1

Physio - PACE

Resp. control	Off
Concatenations	1

Diff - Neuro

Diffusion mode	MDDW
Diff. directions	64
Diffusion Scheme	Monopolar
Diff. weightings	3
b-value 1	0 s/mm²
b-value 2	1000 s/mm²
b-value 3	2000 s/mm²
b-value 1	3
b-value 2	1
b-value 3	1
Diff. weighted images	On
Trace weighted images	On
ADC maps	On
FA maps	On
Mosaic	On
Tensor	On
Noise level	40

Diff - Body

Diffusion mode	MDDW
Diff. directions	64
Diffusion Scheme	Monopolar
Diff. weightings	3
b-value 1	0 s/mm²

Diff - Body

b-value 2	1000 s/mm²
b-value 3	2000 s/mm²
b-value 1	3
b-value 2	1
b-value 3	1
Diff. weighted images	On
Trace weighted images	On
ADC maps	On
Exponential ADC Maps	Off
FA maps	On
Invert Gray Scale	Off
Calculated Image	Off
b-Value >=	0 s/mm²
Noise level	40

Diff - Composing

Distortion Corr.	Off

Sequence - Part 1

Introduction	Off
Optimization	None
Multi-slice mode	Interleaved
Free echo spacing	Off
Echo spacing	0.63 ms
Bandwidth	1894 Hz/Px

Sequence - Part 2

EPI factor	120
RF pulse type	Low SAR
Gradient mode	Performance
Excitation	Standard

Sequence - pTX Pulses

\\USER\CainAkatsuki\Language\L-CA\ep2d_diff_PA
TA: 0:22 PM: FIX Voxel size: 1.6×1.6×1.6 mmPAT: 4 Rel. SNR: 1.00 : epse

Properties

Prio recon	Off
Load images to viewer	On
Inline movie	Off
Auto store images	On
Load images to stamp segments	Off
Load images to graphic segments	Off
Auto open inline display	Off
Auto close inline display	Off
Start measurement without further preparation	Off
Wait for user to start	Off
Start measurements	Single measurement

Routine

Slice group	1
Slices	92
Dist. factor	0 %
Position	L0.0 A7.3 F3.5 mm
Orientation	T > C-5.7
Phase enc. dir.	A >> P
AutoAlign	Head > Brain
Phase oversampling	0 %
FoV read	192 mm
FoV phase	100.0 %
Slice thickness	1.6 mm
TR	3400 ms
TE	77.0 ms
Concatenations	1
Filter	None
Coil elements	HC1-7

Contrast - Common

TR	3400 ms
TE	77.0 ms
MTC	Off
Magn. preparation	None
Fat suppr.	Fat sat.
Fat sat. mode	Weak

Contrast - Dynamic

Averaging mode	Long term
Reconstruction	Magnitude
Measurements	1
Delay in TR	0 ms
Multiple series	Off

Resolution - Common

FoV read	192 mm
FoV phase	100.0 %
Slice thickness	1.6 mm
Base resolution	120
Phase resolution	100 %
Phase partial Fourier	6/8
Interpolation	Off

Resolution - iPAT

Accel. mode	Slice accel.
Accel. factor PE	1
Ref. lines PE	12
Accel. factor slice	4

Resolution - iPAT

Reference scan mode	EPI/separate

Resolution - Filter Image

Distortion Corr.	Off
Prescan Normalize	Off
Dynamic Field Corr.	Off

Resolution - Filter Rawdata

Raw filter	Off
Elliptical filter	Off

Geometry - Common

Slice group	1
Slices	92
Dist. factor	0 %
Position	L0.0 A7.3 F3.5 mm
Orientation	T > C-5.7
Phase enc. dir.	A >> P
FoV read	192 mm
FoV phase	100.0 %
Slice thickness	1.6 mm
TR	3400 ms
Multi-slice mode	Interleaved
Series	Interleaved
Concatenations	1

Geometry - AutoAlign

Slice group	1
Position	L0.0 A7.3 F3.5 mm
Orientation	T > C-5.7
Phase enc. dir.	A >> P
AutoAlign	Head > Brain
Initial Position	L0.0 A7.3 F3.5
R	0.0 mm
A	7.3 mm
F	3.5 mm
Initial Rotation	0.07 deg
Initial Orientation	T > C
T > C	-5.7
> S	0.0

Geometry - Saturation

Fat suppr.	Fat sat.
Fat sat. mode	Weak
Special sat.	None

Geometry - Navigator

System - Miscellaneous

Positioning mode	FIX
Table position	H
Table position	0 mm
MSMA	S - C - T
Sagittal	R >> L
Coronal	A >> P
Transversal	F >> H
Coil Combine Mode	Adaptive Combine
Matrix Optimization	Performance
AutoAlign	Head > Brain
Coil Select Mode	Default

System - Adjustments

B0 Shim mode	Standard
B1 Shim mode	TrueForm
Adjust with body coil	On
Confirm freq. adjustment	Off
Assume Dominant Fat	Off
Assume Silicone	Off
Adjustment Tolerance	Auto

System - Adjust Volume

Position	L0.0 A7.3 F3.5 mm
Orientation	T > C-5.7
Rotation	0.07 deg
A >> P	192 mm
R >> L	192 mm
F >> H	148 mm
Reset	Off

System - pTx Volumes

B1 Shim mode	TrueForm
Excitation	Standard

System - Tx/Rx

Frequency 1H	123.262143 MHz
Correction factor	1
Gain	High
Img. Scale Cor.	1.000
Reset	Off
? Ref. amplitude 1H	0.000 V

Physio - Signal1

1st Signal/Mode	None
TR	3400 ms
Concatenations	1

Physio - PACE

Resp. control	Off
Concatenations	1

Diff - Neuro

Diffusion mode	MDDW
Diff. directions	64
Diffusion Scheme	Monopolar
Diff. weightings	1
b-value	0 s/mm²
b-value	2
Diff. weighted images	On
Trace weighted images	Off
ADC maps	Off
FA maps	Off
Mosaic	Off
Tensor	Off
Noise level	40

Diff - Body

Diffusion mode	MDDW
Diff. directions	64
Diffusion Scheme	Monopolar
Diff. weightings	1
b-value	0 s/mm²
b-value	2
Diff. weighted images	On
Trace weighted images	Off
ADC maps	Off

Diff - Body

Exponential ADC Maps	Off
FA maps	Off
Invert Gray Scale	Off
Calculated Image	Off
b-Value >=	0 s/mm²
Noise level	40

Diff - Composing

Distortion Corr.	Off

Sequence - Part 1

Introduction	Off
Optimization	None
Multi-slice mode	Interleaved
Free echo spacing	Off
Echo spacing	0.63 ms
Bandwidth	1894 Hz/Px

Sequence - Part 2

EPI factor	120
RF pulse type	Low SAR
Gradient mode	Performance
Excitation	Standard

Sequence - pTX Pulses
