# Supplementary material for: The Association Between Changes in White Matter Microstructure and Cognitive Function in Older Adults with Mild Cognitive Impairment
Source: Brain Sci. 2026 Jun 22;16(6):655. doi: 10.3390/brainsci16060655 (PMC13296742; doi:10.3390/brainsci16060655)
Supplement: Supplementary file 1 [file brainsci-16-00655-s001.zip › RESEARCH CONSENT FORM.pdf]

THE SCHOOL OF PSYCHOLOGY, SHENZHEN UNIVERSITY

Human Research Ethics Committee for Non-Clinical Faculties

(RESEARCH CONSENT FORM)

(NAME)		(GENDER)		(AGE)	

(Project Title)	A Study on Brain Networks in Older Adults with Mild Cognitive Impairment: Based on Multimodal MRI Imaging Data

(Identity)	(Name Title)	(Institution)	
(Principal Investigator)		School of Psychology, Shenzhen University	
(Subject Population)	Elderly people		
(Study Contact)			
(Contact phone number)		Email:	

Research Purpose

MCI represents a critical "window period" for the development of cognitive impairment in older adults. However, in China, the assessment tools and diagnostic criteria for MCI have yet to be standardized, and the characteristics of MCI-related brain network changes remain unclear. The lack of multimodal imaging evidence, along with insufficient discussion regarding the correspondence between imaging findings and cognitive performance, has led to contradictions across studies or difficulties in integrating their findings. Therefore, it is necessary to conduct standardized and systematic cognitive function assessments in individuals with MCI and to explore the effects of brain network changes on cognitive function in older adults using complementary information derived from multimodal imaging. Finally, machine learning approaches should be employed to develop improved predictive models for MCI, thereby facilitating a comprehensive understanding of the physiological mechanisms underlying MCI and promoting successful aging.

Procedure

1. Behavioral Experiment

Participants will be required to complete a cognitive test battery, specifically the Cognitive Function Test for Older Adults developed by Beijing Normal University in accordance with the NIA-AA diagnostic criteria. This paper-and-pencil assessment primarily includes an auditory verbal learning test, a clock-drawing test, and the Rey-Osterrieth Complex Figure copy and recall task. The total testing duration is approximately 30–40 minutes. Upon completion, participants' cognitive function status will be evaluated based on their test scores. All information provided will be anonymized, and strict measures will be implemented to ensure data privacy and security.

2. MRI Experiment

Participants who consent to take part in this study will undergo a brain scan using magnetic resonance imaging (MRI) in a quiet, controlled environment. MRI is a non-invasive neuroimaging technique capable of measuring gray matter morphology, white matter fiber networks, and regional changes in blood oxygen level–dependent (BOLD) signals, thereby reflecting brain activity. Prior to the MRI scan, participants will be asked to complete several questionnaires regarding their demographic information, health status, and medication use. During the scan, participants will lie on a specialized bed with their head secured within a circular magnet. They will hear loud, banging noises, which are normal sounds produced during MRI acquisition and should not be a cause for concern. Participants are required to refrain from moving their body, especially their head, throughout the entire scanning procedure. The present study will acquire resting-state data, including structural MRI (sMRI; 7 minutes), functional MRI (fMRI; 8 minutes), and diffusion tensor imaging (DTI; 10 minutes). The total scanning session is expected to last approximately 25 minutes.

Costs

This study will not charge you or your health insurance any fees.

Risks and Side Effects and Benefits

This study does not involve any known serious risks or significant discomfort. However, because MRI scanning involves a strong magnetic field, participants must inform the research team if they have any metal objects or implants (e.g., dental braces, jewelry, pacemakers) either on or inside their body, in order to avoid potential injury or compromise of scan quality. Additionally, participants who have any sensitivity or phobia related to enclosed spaces or loud noises should also notify the research team, who will make every effort to provide a comfortable and safe environment. Participants may find the topics covered in this study to be of personal interest or benefit. They may also gain insight into their own basic cognitive abilities and brain activity through participation in this study.

The rights and obligations of the subject

(1) Rights
If participants experience any physical discomfort or other adverse effects resulting from the experimental procedures during the study, they may notify the experimenter at any time and request to withdraw from the research.
(2) Obligations
Prior to the commencement of the experiment, participants must provide truthful and accurate information regarding their personal physical and mental health status to the experimenter. Any form of concealment or misrepresentation is prohibited. Should any such violation be detected, the experimenter reserves the right to terminate the participant's involvement in the study. During the formal experimental session, participants are required to diligently complete all experimental tasks in accordance with the experimenter's instructions.

Participants must strictly adhere to the experimental procedures as arranged by the experimenter and shall not engage in any behaviors or activities unrelated to the experiment without authorization.

Research confidentiality

This study will strictly protect participants' privacy and data security. All information provided by participants will be anonymized and will not be linked to their identity or contact information. Access to the data will be restricted to members of the research team only. The data will be used solely for the purposes of this study and will not be used for any other purposes or shared with any third parties. When the findings of this study are published, no personally identifiable information of any participant will be disclosed in the article.

Research Voluntariness

Participation in this study is entirely voluntary. Participants may choose to accept or decline the invitation to take part, and may withdraw from the study at any time, without incurring any consequences or penalties.

Subject Statement

I confirm that I have been informed of the purpose, procedures, potential risks and side effects, as well as the anticipated benefits and costs of this study. All of my questions have been satisfactorily answered. I have read and understood this informed consent form in its entirety. My signature below indicates my voluntary agreement to participate in this study. Please note that the signed consent section will not be stored within the study data and will be used solely for the purpose of documenting participant information.

(Signature): (Date):

Experimenter Statement

I have explained the purpose of the study, the study procedures, the potential risks and discomforts, as well as the rights of the participants, and have answered all study-related questions to the best of my ability.

(Signature): (Date):
